# Simulating the psychological and neural effects of affective touch with soft robotics: an experimental study

**DOI:** 10.3389/frobt.2024.1419262

**Published:** 2024-11-29

**Authors:** Caroline Y. Zheng, Ker-Jiun Wang, Maitreyee Wairagkar, Mariana von Mohr, Erik Lintunen, Aikaterini Fotopoulou

**Affiliations:** ^1^ School of Communication, Royal College of Art, London, United Kingdom; ^2^ Division of Media Technology and Interaction Design, KTH Royal Institute of Technology, Stockholm, Sweden; ^3^ Department of Bioengineering, University of Pittsburgh, Pittsburgh, PA, United States; ^4^ Department of Mechanical Engineering, Imperial College London, London, United Kingdom; ^5^ Care Research and Technology Centre, UK Dementia Research Institute, London, United Kingdom; ^6^ Department of Psychology, Royal Holloway, University of London, London, United Kingdom; ^7^ Clinical, Educational and Health Psychology, University College London, London, United Kingdom

**Keywords:** affective touch, CT-optimal touch, affective haptics, soft robotics, S-CAT system, haptic rendering design, stroking touch

## Abstract

Human affective touch is known to be beneficial for social-emotional interactions and has a therapeutic effect. For touch initiated by robotic entities, richer affective affordance is a critical enabler to unlock its potential in social-emotional interactions and especially in care and therapeutic applications. Simulating the attributes of particular types of human affective touch to inform robotic touch design can be a beneficial step. Inspired by the scientific finding on CT-optimal affective touch - a gentle skin stroking at velocities of 1–10 cm/s evidenced to be pleasant and calming, we developed a proof-of-concept haptic rendering system - S-CAT, using pneumatic silicone soft robotic material to simulate the attributes (velocity, temperature and applied normal force) of CT-optimal affective touch. To investigate whether the affective touch performed by the S-CAT system elicits psychological effects comparable to CT-optimal, manual affective touch, we conducted an experimental study comparing the effects of CT-optimal versus non-CT-optimal stimulation velocities in each of three types of stimulation modes (S-CAT device, skin-to-skin manual stroking, hairbrush manual stroking), and across them. Our measures included subjective ratings of touch pleasantness and intensity, neurophysiological responses (EEG), and qualitative comments. Our results showed that velocity modulated subjective and neurophysiological responses in each and across these three stimulation modes, and that CT-optimal stimulations from S-CAT system and manual method received similar ratings and verbal comments on pleasantness, suggesting that the S-CAT touch can have comparable effects to manual stroking. We discuss the design insights learned and the design space that this study opens up to support well-being and healthcare.

## 1 Introduction

Studies have demonstrated that human touch plays a huge role in psycho-emotional and social development with respect to physical and mental health (Varlamov et al., 2020; Gallace and Spence, 2010; Field, 1998; Montagu, 1971). If robotic entities can evoke similar effects to human touch when touching humans, they could potentially provide support to human health. However, it has also been recognised that designing technology-initiated touch in affective human, robot, and virtual-human interactions is a complex endeavour (Ipakchian Askari et al., 2020) and that the current haptic technologies are still very limited in the type of stimulation that they can deliver. There is a lack of haptic actuator design capable of delivering richer stimulation to the skin (Jewitt et al., 2021), and of particular importance is that there is the need for fine-grained modulation of the haptic modalities for creating a sense of natural touch (Olugbade et al., 2023). The development of soft robotics technologies enables design potential for more affective and realistic qualities in touch stimulation through pressurising the skin, with several works suggesting the potential affect-enabling properties of haptic stimulation from such shape-changing mechanism (Hu et al., 2018; Park et al., 2011; Zheng, 2018).

One particular type of affective touch, namely gentle, slow-moving, caress-like touch, especially when moving across the skin at a velocity of 1–10 cm/s is found to be most affective (Ackerley et al., 2014a; Löken et al., 2009). This type of affective touch is referred to as CT-optimal affective touch, as it most favourably excites the C-tactile (CT)-afferents and correlates strongly with reported pleasantness (Löken et al., 2009). CT-optimal touch delivered by a soft brush has shown to be beneficial in alleviating stress (Triscoli et al., 2017), pain (Krahé et al., 2016; Liljencrantz et al., 2017), and the sense of social isolation (von Mohr et al., 2017). Despite the evidenced benefit of CT-optimal touch for socio-emotional interactions, there is still a scarcity of haptic systems exploiting physical properties for affective touch interactions, with the exception of Ipakchian Askari et al., 2020, Dekker et al. (2021) and Zhao et al. (2023). Haptic devices in this work simulate the velocity of a CT stroking using laterally moving stimulators driven by a stepper or DC motor. Utilising the naturalistic and affective effects of soft robotic actuators towards creating high-resolution device for delivering CT-optimal touch is still unchartered. In addressing this gap, we developed a high-resolution soft robotic haptic rendering system that aims to deliver CT-optimal affective touch (hereafter referred to as the S-CAT system) through simulating the physical attributes of a CT-optimal touch in terms of velocity, normal force and temperature. We designed a lab-based study to experimentally assess the validity of the soft robotic S-CAT device in producing tactile stimulation that has psychological effects comparable to CT-optimal affective touch, as delivered manually either by skin-to-skin contact or via a brush. In this study we compared these three types of CT-optimal stimulation with CT-optimal and non-CT-optimal stimulations elicited with each of the respective methods.

The original contribution of our work includes: 1) demonstrating a novel design of a soft robotic, affective touch rendering system simulating the velocity, temperature, and force of gentle stroking, 2) evidencing that a soft robotic actuator is capable of delivering CT-optimal touch, and can elicit comparable psychological effect as when manual touch is applied, and 3) composing a design space for applying CT-optimal touch delivered by robotic entities, which include design insights and ethical considerations for machines to mimic human touch. In existing work, when evaluating touch stimulations from stimulators such as soft brush and haptic devices, most often only the stroking at CT-velocities and non-CT-velocities from the same stimulator were compared. In this paper, we not only compared the effects of stroking at CT-optimal versus non-CT-optimal velocities applied by each of three types of stimulation modes (robotic device, skin-to-skin manual stroking, brush manual stroking), but also across them, thus providing additional insights comparing different types of touch stimulators.

In the next section, we elaborate on relevant literature on CT-optimal affective touch, the space of designing affective touch initiated from technologies and the affective qualities of soft robotic actuators. We then present the design and evaluation of the S-CAT system. We discuss the insights learned from this study, including the affective affordance of the S-CAT system, design insights on simulating human affective touch to higher fidelity, the design space this study opens up to support health and wellbeing.

## 2 Relevant work

### 2.1 CT-optimal touch

Inter-personal touch is an important dimension to facilitate mental and physical wellbeing. There has been increasing scientific and design interest in a particular type of affective touch, namely gentle, slow-moving, caress-like touch (e.g. Löken et al., 2009; Fotopoulou et al., 2022). In particular, the kind of caress that satisfies the following parameters: moving across the skin at a velocity of 1–10 cm/s (Ackerley et al.,, 2014b; Löken et al., 2009), an indentation force between 3 and 0.25 mN, with temperature similar to skin temperature (Ackerley et al., 2014a) is found to be most affective, as it optimally excites the C-tactile (CT)-afferents and correlates strongly with reported pleasantness (Löken et al., 2009). This type of caress is also called CT-optimal touch. CT afferents are unmyelinated nerve fibres conducting at slow speed. They are widely evidenced to be present on hairy skin, although recent research has shown that they may also be present to a lesser degree in non-hairy skin such as the palm (Watkins et al., 2020). CT afferents are thought to project via thalamic pathways to brain regions correlated with emotion-related processing, social cognition, and interoception, such as the insular and orbitofrontal cortices (Björnsdotter et al., 2010; Bud Craig, 2009; Gordon et al., 2013; Olausson et al., 2002). In contrast, fast touch, which serves a discriminative purpose is thought to be mediated mainly by a different type of nerve fibres - Aβ afferents and to be processed in the somatosensory cortex (Liljencrantz and Olausson, 2014). The Aβ afferents stimulating touch with greater velocities is known to lead to a linearly enhanced perception of tactile intensity, while there is no such linear relationship with perceived pleasantness (Case et al., 2016; Löken et al., 2009; Sailer and Ackerley, 2017).

Several lines of research in affective touch, including the literature on CT afferents, have noted the beneficial role of touch throughout the human lifespan (Fotopoulou et al., 2022 for review). For example, skin-to-skin touch is associated with calming, soothing effects in infancy (Field, 2014) and promotes bonding in early parental interactions (Hofer 1994). CT-optimal touch delivered by a soft brush has shown to be beneficial in modulating pain in adults (Fotopoulou et al.,, 2022), eliciting positive affective valence (e.g. Pawling et al., 2017; Perini et al., 2015), alleviating stress (Triscoli et al., 2017), and reducing the sense of social rejection (von Mohr et al., 2017). Tactile-based interventions were also found to improve clinical outcomes, such as helping with pain management for infants in neonatal intensive care units (NICU) (Hathaway et al., 2015: for review, see Field, 2014). In some studies, CT-optimal touch was delivered manually, via a brush e.g., while in others by a robotic device that could control the brush strokes (e.g. Ackerley et al., 2014a; Ackerley et al.,, 2014b; Löken et al., 2009; McGlone et al., 2014; Triscoli et al., 2017).

### 2.2 Affective touch initiated by technologies and the affective affordance of soft robotic actuators

Driven by the critical role in health and wellbeing, and the social benefit of inter-human affective touch, the haptic and design community has explored different types of haptic actuators contributing to different affective affordance of the touch stimulation produced. Traditionally, vibratory actuators are the most frequently used to produce haptic stimulation (Huisman et al., 2016). However, there is limited scope for using vibratory actuators to simulate human touch, particularly since that it is not capable of replicating the pressure of human touch (Rossiter et al., 2017). There are also significant limitations for sensations from high-frequency vibratory actuators to elicit affective responses. Rather than evoking pleasant feelings, research has shown that their high-frequency movements can induce negative sensations after lengthy exposure (Kaaresoja and Linjama, 2005). There is an aspiration from the design community to move beyond vibrotactile applications to make technology-initiated touch a richer experience (Jewitt et al., 2021). In exploring touch stimulators that bear higher affectivity, design researchers have been exploring alternative actuators, including heat (*Anon, 2014*), shape-changing interface (Gupta et al., 2017), friction (Bianchi et al., 2016; Wang et al., 2010; Zhao et al., 2023), mid-air (Obrist et al., 2015; Tsalamlal et al., 2018), and soft robotics (Hu et al., 2018; Teh et al., 2008; Zheng, 2018). Compared to other actuators, soft, pliant pneumatic actuators inspired by soft robotic principles (Ilievski et al., 2011) have shown a closer resemblance of features of human touch. They are actuated by pressure through shape-changing movements. The surface texture of soft, pliant material such as silicone bears a significant resemblance to human skin. The force-applying mechanism also resembles the way the human hand applies force (Rossiter et al., 2017). Single pneumatic actuator with 1 degree of freedom (DOF) has been explored with to represent human affective touch gestures such as squeezing, hugging (Teh et al., 2009), poking, or tapping (Park et al., 2011; Wang and Zheng, 2019), and the touch stimulations from these soft actuators have been reported to be perceived as more human-like, emotionally resonant and pleasant (e.g. Park et al., 2011; Zheng, 2018), promoting calming sensations (Tsaknaki, 2021) or mindfulness (Jung et al., 2021). Articulating multiple soft actuators with the aim to produce more sophisticated affective touch gestures such as CT-optimal stroking in forms of high fidelity, simulating the velocity, temperature, and normal force has been under-explored.

In the field of Human-robot Interaction (HRI) design, the humanoid robotic hand is the key conductor when the robot is conveying affective emotion to a human through touch (Olugbade et al., 2023). However, studying and designing interactive robotic hands requires tackling more complex questions and challenges (Piazza et al., 2019). Apart from humanoid robotic hands, a robotic device with a haptic rendering surface can also be used to transfer affective experience, simulating the feeling of being touched (Olugbade et al., 2023; Huisman et al., 2013). Compared with a humanoid robotic hand, a haptic rendering system is more agile, versatile, and re-configurable. Soft, pliable actuators can be attached to robot arms, social robotic entities, body-contacting interfaces such as chairs and seats and inside of clothing to deliver an affective touch. For example, Hu et al. (2018) developed a soft texture-changing skin with goosebump and spike units as the outside textures of a social robot, using pressurised elastic materials; Wang and Zheng (2019) developed a soft robotic rendering system enclosed into a textile wristband, that mimics human affective touch with the intention to build social bonds and regulate emotional and cognitive functions. Thus, we are motivated to create such a versatile haptic rendering system, that can be potentially integrated into both wearable and social robotic systems aiming for affective interaction.

One can take at least two different approaches to create technology-mediated touch. In one approach, one can try to find technological means to generate stimuli that mimic the sensory properties of natural touch stimuli, but given the complexity of the human tactile sensing system and of the social contexts in which it occurs, this ‘emulation’ approach has been found to be complicated (Ipakchian Askari et al., 2022). A stroking mechanism that both applies normal force and has lateral movements across the skin, requires a bulky and complex device design, as in Askari et al. (2023), Dekker et al. (2021) and Zhao et al. (2023). The haptic rendering system that we designed in this study, focuses on a complete soft and flexible solution, taking advantage of the naturalistic effect of silicone material pressurising the skin through pneumatic actuation. Instead of building a bulky, motor driven mechanism to apply a lateral movement for the stroking, the actuators within the S-CAT system applies normal force in close distance consecutively, exploiting the “haptic illusion” effect of a stroking movement across the skin (Geldard and Sherrick, 1972; Seizova-Cajic and Taylor, 2014) (more detail of the actuation in Section 3). The approach we have taken here is an alternative approach to the “emulation” approach, in which we strive to find technological means to generate the functions of touch, particularly the subjective, emotional effects of affective touch stimuli. The notion of “extending” the mind by technology is well-developed notion in philosophy, psychology, and other fields (Clark and Chalmers, 1998). In the same way, as clothes act as a kind of “second skin” by extending their protective and thermoregulatory functions, our approach aims to technologically “extend” the psychosocial functions of CT-optimal touch and hence ultimately recreate its human values in digital and mediated communication. Hence, in this first study we wish to test if the affective effects of our device are comparable to that of CT-optimal touch.

## 3 The S-CAT haptic rendering system

The S-CAT haptic rendering system is a soft robotic system that aims to deliver CT-optimal affective touch (S-CAT). In order to explore the most affective touch quality, in addition to the velocity of CT-optimal touch, which is between 1 and 10 cm/s (Löken et al., 2009; Mountcastle, 2005), we also incorporated additional physical traits of temperature and applied normal force into our design exploration. Literature on CT-optimal touch suggests that an approximately skin temperature produces the highest CT firing frequencies (Ackerley et al., 2014a). Warmth in physical contact from social robots can be perceived as more enjoyable (Block and Kuchenbecker, 2019). We have collected available information on the force applied in studies evaluating CT-optimal touch, which shows a range of applied normal force between 0.2 and 0.5N (Ackerley et al., 2014a; Manzotti et al., 2019; McGlone et al., 2007; Pawling et al., 2017; Vallbo et al., 1999). Although neither of the previous work such as (Bianchi et al., 2016; Zhao et al., 2023) reported on the noise factor affecting the affective affordance of the touch, from our preliminary studies we have learned that the noise level from the electronic parts contributes negatively to the perceived pleasantness toward the affective touch stimuli. Thus we made an effort to incorporate noise reduction in our design.

**FIGURE 1:**
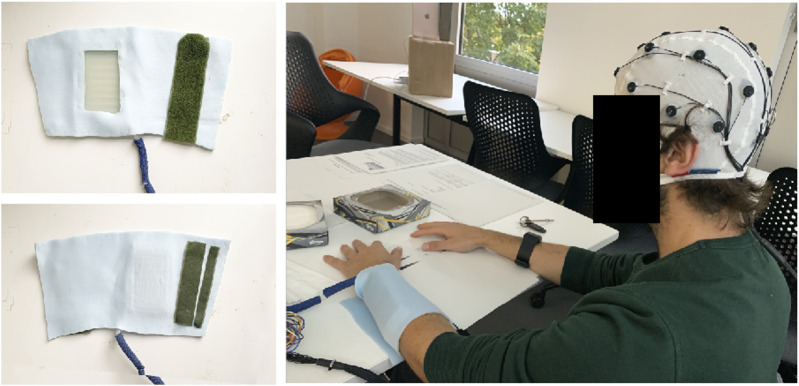
The S-CAT band. Image on top left shows the side that contacts the skin. Image on the right shows how it is worn on the arm during stimulation sessions.

**FIGURE 2:**
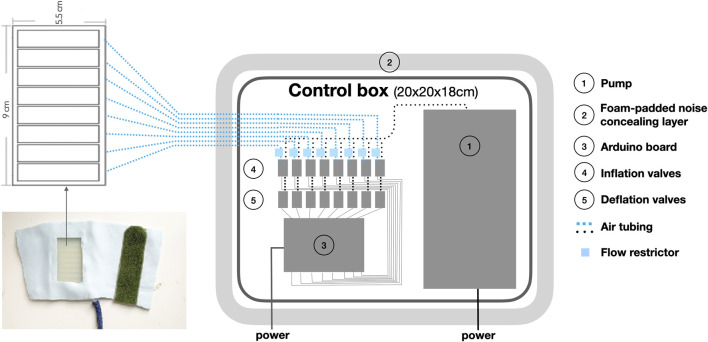
Dimension of the actuator design (top left) and the control units in the control box.

The S-CAT system (Figures 1, 2) is a proof-of-concept prototype for a completely soft, wearable affective touch stimulator that is easily configurable. It is a refinement of the prototype used in a previous study (Wang and Zheng, 2019). It consists of an armband (Figure 1, left) and a control box (Figure 2). The armband is completely soft, with no hard components. The actuator is an array of 8 individually controlled pneumatic cells that are arranged with minimum spacing as shown in Figure 2 (left). The actuator is cast from an in-house designed mould and is made from Ecoflex™ 00-35 FAST silicone material. The dimensions of the surface of the actuator are 9 cm long and 5.5 cm wide, and when inflated, the skin-contacting area of the robot stimulating surface is around 4 cm wide and 9 cm long. When the individual cells are actuated in a sequence with an overlap, a tracing effect is produced to simulate a caressing touch gesture. The stroking velocity is the speed with which the sensation of the touch moves across the body of a user. The stroking velocity is the rate of propagation of the individual cell being inflated and thus pressing upon the body, one point after another. The stroking velocity (v) can be calculated by using the distance between the centres of two adjacent cells (d) divided by the time lag between the inflation of two adjacent cells:

$$T_{1}:v=d/T1$$

The width of each actuator, and the control of the array of cell behaviours are coordinated to achieve the best continuity: that is, the rippling of the two adjacent cells contacting the skin will not be perceived as two touches but more as one continuous tracing. It is not possible to eliminate the gap between two separate ripples using this actuation method, no matter how close the adjacent cell is (d) and how short the interval between the actuation of the adjacent cell (T_1_). However, according to research, minimal interruption in the continuity of touch can be made up by human perception, which is also referred to as haptic illusion (Geldard and Sherrick, 1972; Seizova-Cajic and Taylor, 2014). The approach of using a sequence of normal force indentations that create the ‘illusion’ of a stroking sensation has been also explored by Huisman et al. (2016) using vibrotactile actuation, Nunez et al. (2020) and Culbertson et al. (2018) using voice coil actuators as well as by Muthukumarana et al. (2020) using Shape Memory Alloy actuator, however with larger distance between adjacent indentation points. We have achieved a minimal distance between adjacent indentation points of 12 mm, comparing to 30 mm in (Huisman et al., 2016), 33 mm in (Culbertson et al., 2018), and 25 mm in (Nunez et al., 2020).

The pneumatic valves (Figure 2), the electronic micro-controller (Figure 2), and the power are contained in a control box external to the band, connected only via approximately 2 m-long air tubing to each of the actuators within the band. In terms of configurability, the S-CAT device can perform adjustable stroking speeds between 6 cm/s ∼ 36 cm/s through coding. For velocity slower than 6 cm/s, the perceived continuity began to be compromised, and for velocity faster than 36 cm/s the perception of directionality starts to be unclear. We adopted an applied normal force of 0.48N that applies to the skin. For noise reduction, we have chosen the type of air compressing that generates the least noise (Schego optimal, Nr.850, 220–240 V/50 Hz – 5w, Figure 2), and have used a foam padded case as an isolation container to conceal the remaining noise. The silicone actuator part of the armband was kept on top of a box in warm water with the temperature controlled at 36.5°C to maintain the consistency of body temperature.

## 4 Materials and methods

### 4.1 Study design and hypothesis

To evaluate if the S-CAT design can elicit psychological effects comparable to CT-optimal affective touch delivered by soft brush or skin-to-skin touch, we conducted a lab-based study in which we compared tactile stimulations delivered in a CT-optimal velocity versus a non-CT-optimal velocity by all these three methods. To quantify the subjective perception of these classes of stimuli, we sampled subjective ratings of the perceived pleasantness and intensity of each stimulation on visual analogue scales as in various previous protocols (Pawling et al., 2017; Sailer and Ackerley, 2017; Case et al., 2016; Gentsch et al., 2015; Jenkinson et al., 2020). Given the aforementioned stimulation profile of CT and Aβ fibres, we expected tactile stimulation delivered at slower, CT-optimal speeds to be associated with increased pleasantness compared to those delivered at faster, non-CT-optimal velocities, irrespective of stimulation method. The reverse was expected regarding intensity ratings, again irrespective of stimulation method (Case et al., 2016; Sailer and Ackerley, 2017). This pattern of results, together with the absence of any other significant differences in pleasantness ratings between the three stimulation methods, would signify that the new S-CAT haptic device is capable of eliciting different psychological effects from CT-optimal touch in comparison to non-CT-optimal velocities, as in the case of either skin-to-skin stroking or via a brush. To explore these comparisons further, we also collected qualitative comments in response to a brief open question, administered after the tactile stimulation, as existing qualitative studies have been informative regarding soft robotics and affective touch. Finally, to explore individual differences in response to touch delivered by the S-CAT device and other methods, we also administered a validated questionnaire that assesses preferences and attitudes to social touch in everyday life. We anticipated a lower differentiation between CT-optimal and non-CT-optimal touch in people with generally more negative attitudes to social touch, irrespective of stimulation method.

In addition to the above subjective measures, we also explored the potential of using neurophysiological response with electroencephalography (EEG) to quantify neural responses to the tactile stimulation of the device, as well as to manual touch. To our knowledge, only a handful of studies have assessed neurophysiological responses to affective touch; preliminary research examining a single infant participant evidenced increased theta activity in posterior leads, in response to maternal affective touch stimulation (Maulsby, 1971). In another study, haptic stimulation by fabric within CT-optimal velocity is associated with alpha/beta suppression, particularly in the regions covering somatosensory areas (Singh et al., 2014). Conversely, enhanced theta activity has been found in response to Aβ-discriminative touch (also accompanied by a decrease in alpha activity), with theta activity correlating with subjective ratings of stimulus intensity. (Michail et al., 2016; Valenza et al., 2015) applied combinations of two forces (2N and 6N) and three velocities (9.4 mm/s, 37 mm/s, and 65 mm/s) to the forearm (stimulating the area across the width of the forearm) of participants and found that the more the affective stimuli become unpleasant the more the brain dynamics desynchronised across all regions of the scalp. In a more recent study, affective touch stimulation within CT-optimal velocity using a soft brush on the forearm was found to be associated with decreased theta activity across the scalp and decreased beta activity in parietal areas, compared to conditions of rest and when stimulated with nonaffective touch (non-CT-optimal velocity) (von Mohr et al., 2018). There are limited studies on the effects of affective touch on neurophysiological responses available in the literature and there is a lack of consensus on the results. Hence, based on results from von Mohr, Crowley et al. (2018), in this study we explore the effects of affective touch delivered by different types of stimuli (a robotic device, a brush, and human touch) on EEG oscillations in different frequency bands. We hypothesise a decrease in theta and beta activity in response to tactile stimulation versus rest and in response to CT-optimal velocities in comparison to non-CT-optimal velocities, particularly in temporal and parietal regions, across all three stimulation modes.

In summary, to explore the validity of the soft robotic S-CAT system in producing psychological effects comparable to CT-optimal affective touch delivered by soft brush or skin-to-skin touch, we compared tactile stimulations delivered in a CT-optimal velocity versus in a non-CT-optimal velocity by all these three methods. We collected quantitative, subjective ratings on touch pleasantness and intensity, neurophysiological responses (EEG), and qualitative comments. If the soft robotic S-CAT device is capable of eliciting comparable psychological effects to human touch, our hypothesis was constructed as follows:

H1: Our participants would give on average higher pleasantness ratings and lower intensity ratings in response to slow touch compared to fast touch in all three stimulus types, with no differences between the type of stimulation (human, robot, brush), or in interaction with velocity.
H2: Decreasing EEG band power, particularly in the theta and beta frequencies in temporal and parietal areas, when comparing touch against rest and affective, CT-optimal touch against non-CT-optimal touch in all types of stimulation separately (human, robot, brush) based on previous study (von Mohr et al., 2018), with no differences between the type of stimulation (human, robot, brush), or in interaction with velocity.
H3: We also collected brief qualitative comments to further enhance our understanding of the above hypotheses and exclude any adverse or unexpected effects such as fear or disgust towards the soft robotics device.

### 4.2 Participants

Twenty-two participants (13 female, 8 male, 1 prefer not to say, age mean 29.5, age SD 9.6) were recruited at the Royal College of Art, without any prior knowledge of the device or the relevant touch literature, or the study’s aims and hypotheses. This sample size was determined based on power calculations using GPower 3.1 based on a previous study on EEG power and CT vs. non-CT optimal touch using a brush (von Mohr et al., 2018) with an effect size of η^2^_partial=_.18. The Royal College of Art Research Ethics Committee approved all the procedures prior to recruitment, and all participants provided written informed consent. No compensation was involved in participation.

### 4.3 Conditions

We employed a 2 (tactile speed: slow vs. fast) x 3 (stimulation type: human skin, brush, robot) within-subjects design; hence six experimental conditions: human slow (HS), human fast (HF), brush slow (BS), brush fast (BF), robot slow (RS) and robot fast (RF). A slow tactile stimulation speed of 6 cm/s was chosen because this is within the optimal range for targeting CT afferents (Löken et al., 2009) and it is also the slowest speed of continuous touch that the soft robotic S-CAT device can perform. A fast tactile stimulation speed of 36 cm/s was chosen for this is a non-CT-optimal speed. No studies comparing CT-optimal touch generated by robot, via a brush and skin-to-skin have been conducted before and we wanted to assess the performance of the S-CAT device in comparison to the well-known affective touch-generating methods of hand and brush.

**FIGURE 3:**
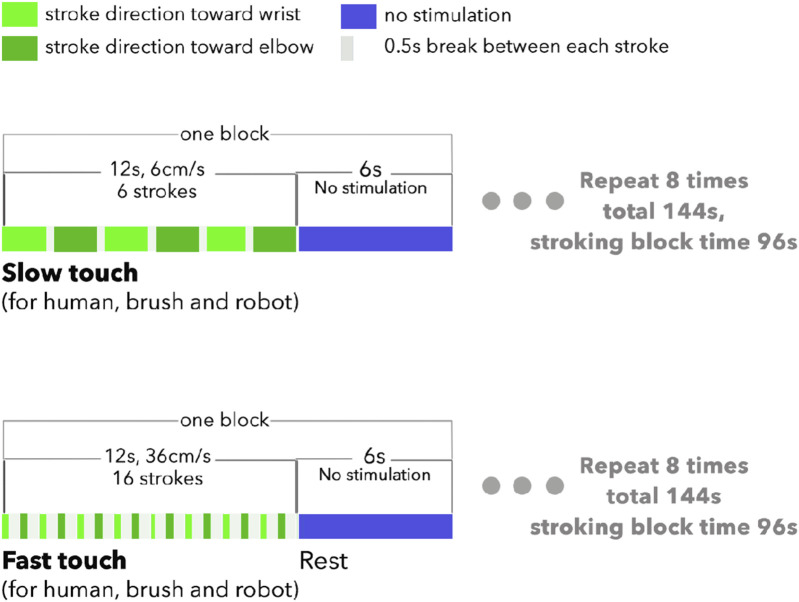
Design of slow (CT-optimal speed) touch and fast (non-CT-optimal speed) touch stimulation sessions.

The order of the experimental conditions was randomised across participants. Each experimental condition consisted of eight blocks: each block had 12 s of tactile stimulation followed by 6 s of rest (see Figure 3). The tactile strokes were applied back and forth in the stimulation area, with a break of 0.5 s between each stroke. The break between each stroke was intended to minimise habituation (McGlone et al., 2007). Outcome measures were (a) subjective ratings of perceived pleasantness and intensity/activation following tactile stimulation after each experimental condition and (b) EEG activity across frequency bands, namely delta, theta, alpha, beta, and gamma.

### 4.4 Stimulation types other than the S-CAT system

#### 4.4.1 Human skin

A female experimenter kept her four fingers together and used the area between the middle phalanx and the proximal phalanx of her left hand as the skin-contacting area. This is because when experimenting with human hand touch during the design phase, applying human touch using this area had shown consistency in covering the 4 cm-wide marked stimulation area on the arm, and also because compared with using the palm of the hand, the force applied using this gesture appeared to be more controllable and it produced the most favourable consistent force (see also Gentsch et al., 2015). As with the robot stimulator, the experimenter placed her hand on top of a box in warm water with the temperature controlled at 36.5°C to maintain the consistency of body temperature.

#### 4.4.2 Brush

As in previous studies (e.g. Crucianelli et al., 2013; Krahé et al., 2016; von Mohr et al., 2017), we used a soft brush (No.7 natural hair blusher brush, The Boots Company) held by the same experimenter delivering the skin-to-skin touch in the above conditions, to deliver tactile stimulation manually at slow (6 cm/s) and fast (36 cm/s) velocities.

### 4.5 Subjective pleasantness and activation/arousal reports

**FIGURE 4:**
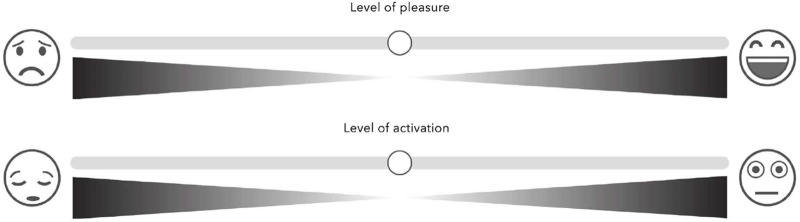
Adaptation of Affective Slider (von Mohr et al., 2018; Voos et al., 2013), divided by 100 points.

For the behavioural data, we used the AffectiveSlider (Betella and Verschure, 2016) (Figure 4) for participants to give their subjective rating of the level of felt pleasantness and the level of activation (intensity of felt arousal) following tactile stimulation after each experimental condition. We explained to the participants that “the pleasure scale refers to the level of pleasantness that you feel about the sensation, the furthest left position being the most unpleasant and the furthest right indicating extremely pleasant,” and “the scale of activation refers to how activating or arousing, or how intensely you feel, the sensation is, the furthest left position indicating the least activating and the furthest right indicating extremely activating”. The scale ranged from 0 “not at all” to 1 “extremely”.

#### 4.5.1 Qualitative reports

Immediately after each touch stimulation session, we asked a single open question, “How did that make you feel?” to allow participants to freely describe verbally their subjective feelings. In addition, participants were also asked to comment on the perceived continuity of the slow touch from the S-CAT device, on a scale of 0–10.

#### 4.5.2 Social Touch Questionnaire

Affective touch is a form of social touch: people’s pre-existing attitude toward social touch may influence their perception of affective touch (Krahé et al., 2018; Wilhelm et al., 2001). For this reason, we also collected information on individual preferences relating to touch using a Social Touch Questionnaire (STQ) (Wilhelm et al., 2001). The STQ (provided as Supplementary Material) is a self-reporting measure that assesses participants’ attitudes toward social touch and has been employed in previous studies on CT-optimal touch (Bennett et al., 2014). Scores range from 0 to 80; higher scores indicate an aversion to giving, receiving, and witnessing social touch. We aimed to explore whether there are individual differences in how people respond to touch from the S-CAT device based on their preferences regarding touch.

#### 4.5.3 EEG recording and data processing

**FIGURE 5:**
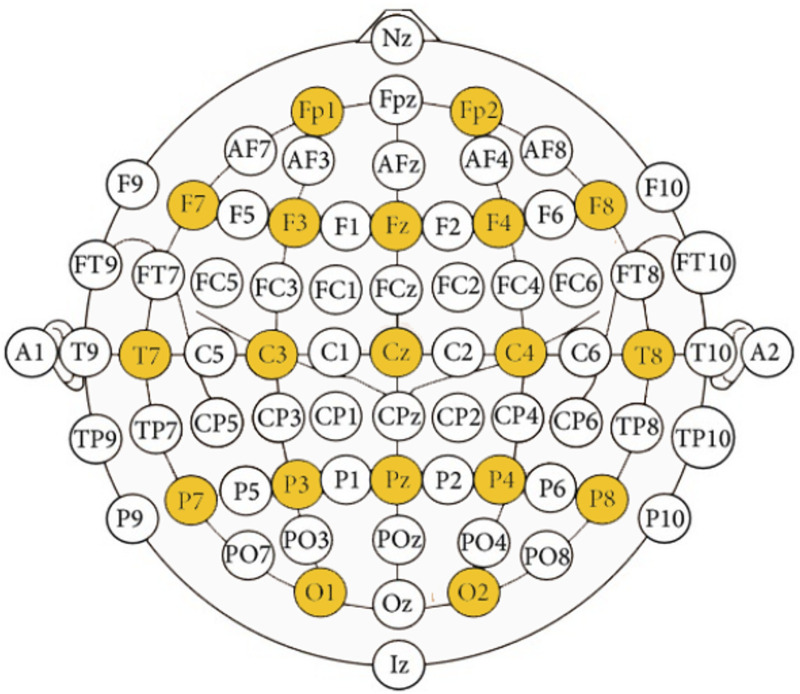
Placement of 19 electrodes (highlighted in yellow).

For collecting EEG data, we used an EEG Electrode Cap Kit from OpenBCI (OpenBCI, New York, United States), with the application of wet electrodes (Ag/AgCl coated). We recorded EEG signals using OpenBCI Cyton + Daisy biosensing board (16 Channels, 24-bit data resolution, 125 Hz sampling rate). The performance of the OpenBCI EEG capturing system has also been tested in existing studies e.g. (Lakhan et al., 2019). The placement of electrodes is shown in Figure 5.

EEG data from 22 participants was collected. During data inspection, data from 11 participants were of satisfactory quality. We rejected data from 11 participants with low-quality EEG signals due to prominent movement artefacts, low signal-to-noise ratio, inconsistent time-frequency electrode response, and noisy channels due to potentially unstable electrode/wire connections with either human skin or the electric board. The raw EEG data were pre-processed with a band-pass filter at 0.5 ∼ 50 Hz, to eliminate DC offset drifts and the high frequency and power line noise. The EEG time-series data were then segmented using the sliding window (1 sec, 125 samples), and the Fast Fourier Transformation (FFT) technique, to obtain the power spectrum of the signal to be analysed in the frequency domain. We extracted the average peak values of the power of each frequency band–Delta (0.5–4 Hz), Theta (4–8 Hz), Alpha (8–13 Hz), Beta (13–30 Hz) and Gamma (30–80 Hz), as the features to be analysed. These features were further smoothed out by an averaging filter (with a 2-sec sliding window) over time to remove eye movement, blinking, and muscle artefacts. The entire EEG data processing and analysis was conducted in MATLAB R2018a (The MathWorks Inc., Natick, United States) and our custom scripts, as well as the EEGLAB toolbox (Delorme and Makeig, 2004).

### 4.6 Procedure

The study was held in a designated quiet room, with plain white walls. There were two researchers (female and male) present in the room during every testing session.

First, the experimenter marked an area of 9 cm in length and 4 cm in width on the hairy side of the left forearm of each participant, with a water-removable marker pen, to identify the area where all the touch stimuli would be applied, as in (Crucianelli et al., 2013; Krahé et al., 2016; von Mohr et al., 2017). Following this, participants were introduced to the three means of stimulation: hand, brush, and the S-CAT device as an autonomous device. Participants then closed their eyes to experience briefly all six experimental conditions that were going to be assessed in the study.

Prior to the experimental tactile conditions, each participant was asked to close their eyes and relax for 5 min (300 s). EEG data about this resting status was collected as a baseline. The participant then opened their eyes and flexed their muscles before the first touch stimulus was applied. Next, to make sure that there would not be a big temperature drop, before applying to the skin, the robot touch device and the human hand were put on the above-mentioned warmer for approximately 5 min to be kept at approximately body temperature. All the manually applied touch stimuli (from human hand and brush) for all the participants were administered by the same trained female experimenter throughout the study. The experimenter was presented with an on-screen visual guide during each manually applied touch condition to facilitate consistency in the velocity of brushing and hand touching (as in von Mohr et al., 2018; Voos et al., 2013). To apply stimulations from the S-CAT device, the experimenter first helped put the sleeve-like soft band of the device around the participants’ forearm and made sure the surface area of the actuator covered the marked stimulating area of the skin. The experimenter then triggered a button (not visible to the participants) that will activate the stroking pattern at either slow or fast speeds.

Participants were asked to close their eyes during the experimental conditions, including the tactile and rest blocks. The experimenter checked during the stimulation and ensured that they all had their eyes closed throughout. Thus, although participants saw the three types of stimulators prior to the study tasks, they did not see which of the six experimental conditions were applied at the time of receiving each stimulation. After each condition, participants were asked to open their eyes, rate the perceived pleasure and activation/arousal using the AffectiveSlider, and verbally respond to the open question.

Participants were asked to close their eyes as a reset for 40–60 s before the next stimulus to avoid a carry-on effect from previous stimuli and to help return to neutral/calm status. The experimental session lasted approximately 40 min.

### 4.7 Plan of data analysis

#### 4.7.1 Pleasantness and arousal subjective ratings

We first examined descriptive statistics, followed by inferential statistics. Specifically, we conducted repeated measures of ANOVAs specifying stimulation type (human, brush, robot) and speed (slow, fast) separately on pleasantness and activation/arousal ratings. Effect size is presented as partial eta-squared (η2partial), where .01 represents a small effect size, .06 represents a medium effect size, and .14 represents a large effect size (Cohen, 1988). Greenhouse-Geisser corrections were used when sphericity assumptions were violated. In addition to our main behavioural analyses on pleasantness, we conducted planned contrasts (two separate t-tests; using Bonferroni adjusted alpha levels of 0.025 per test) on pleasantness rating for slow robot touch against slow brush touch and separately against slow human touch, as these were the critical comparisons regarding the device’s potential for eliciting pleasantness ratings comparable to those of human-to-human CT-optimal touch. In addition, based on our expectation about the possibility of the robotic device leading to comparable pleasantness effects to human or brush touch, non-significant (p> .025) findings in these planned contrasts were followed up using Bayesian statistics (Carey et al., 2021), which present the ratio of the likelihood of the alternative hypothesis relative to the likelihood of the null hypothesis. A Bayes Factor (BF) > 3 indicates evidence for the alternative hypothesis, whereas a BF < 0.3 indicates evidence for the null hypothesis. A BF between 0.3 and 3 indicates an inconclusive result which is not in favour of either hypothesis. This is possible for both parametric and non-parametric hypothesis testing (van Doorn et al., 2020).

#### 4.7.2 EEG data

To observe the effects of different types of stimuli (fast and slow strokes applied by hand, brush, and robot) and a resting condition on band power of different frequencies in different brain regions, we first examined descriptive statistics by tabularising mean and standard deviation of band powers of all 11 participants. Due to the limited amount of data, we then used separate non-parametric Friedman tests to compare changes in theta and beta frequency bands in parietal and temporal regions independently (von Mohr et al., 2018) between all the six different types of tactile stimulation and rest. We used resting state EEG as a baseline where no tactile stimulus was presented. The significance threshold was set to *p* = 0.05. For cases where the *p*-value from the Friedman test indicated statistical significance, pairwise comparison was done for each of the stimulus conditions using Dunn-Bonferroni *post hoc* tests specifically to compare 6 touch stimuli against the resting condition. Next, we compared slow touch against fast touch in all three types of stimulation separately, using the Wilcoxon Signed Ranks Test to observe the difference between CT-optimal and CT-sub-optimal touch. Finally, to compare three modes of touch stimuli, we compared band powers of slow human, slow brush, and slow robot touch using separate Friedman tests in theta and beta bands in temporal and parietal regions.

#### 4.7.3 Qualitative comments

Participants’ open-ended comments were recorded and transcribed. Initial recordings were deleted after transcription. The data was analysed by one researcher, applying a reflexive thematic analysis approach to code and uncover patterns across the data (Braun and Clarke, 2021; 2022). We adopted an inductive-experiential orientation (Braun and Clarke, 2022) taking the view that the language expressed by the participants offers direct insights into their subjective felt experience as touch receivers. The initial step was for the researcher to familiarise with the recordings and the transcription text. Next, comments for each of the six experimental conditions from all participants were coded and comments from each single participant regarding all six experimental conditions were also coded. These datasets and the initial codes were then reviewed and the initial codes were refined or clustered to form a second version of the codes. Following this, general themes were identified and discussed among the project team, before the final themes were formulated.

## 5 Results

### 5.1 Pleasantness and arousal subjective ratings

**FIGURE 6:**
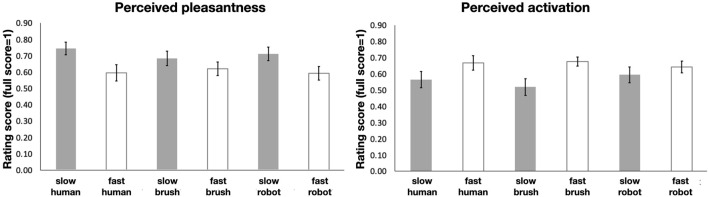
Subjective mean rating on pleasantness rating (left) and activation rating (right). Error bars denote standard error of the mean.

Analyses of pleasantness ratings showed that the main effect of speed, *F* (2,21) = 9.06, *p* = .007, η^2^_partial=_.30, was statistically significant. Averaging across stimulation type, slow touch (M = .71, SD = .15) was reported as more pleasant than fast touch (M = .60, SD = .13). The main effect of stimulation type was non-significant, *F* (2,42) = .12, *p* = .887, η^2^_partial=_.01, and stimulation type did not interact with speed, *F* (2,42) = .93, *p* = .402, η^2^_partial=_.04. Thus, participants perceived slow vs. fast touch as more pleasant, irrespective of stimulation type (i.e., robot, brush or human touch). See Figure 6, left panel.

Similarly, analyses of activation/arousal ratings showed that the main effect of speed, *F* (2,21) = 6.27, *p* = .021, η^2^_partial=_.23, was statistically significant, with fast touch (M = .71, SD = .15) perceived as more arousing/intense than slow touch (M = .60, SD = .13). The main effect of stimulation type was non-significant, *F* (2,42) = .17, *p* = .820, η^2^_partial=_.01, and stimulation type did not interact with speed, *F* (2,42) = 1.70, *p* = .194, η^2^_partial=_.08. Thus, as expected and in contrast to perceived pleasantness, people perceive fast vs. slow touch as more arousing or intense, irrespective of stimulation type (i.e., robot, brush or human touch). See Figure 6, right panel.

There was no significant difference in our planned contrast between the pleasant rating of Robot slow (M = .71, SD = .20) vs. Brush slow touch (*M* = .68, *SD* = .21; t (42) = 0.53, *p* = .604)). We then conducted Bayesian analysis, testing the null hypothesis against the alternative hypothesis that robot touch may lead to less pleasantness feelings than brush touch. We obtained a result of BF_10_ = 0.157 (error ∼8.162e-4, assuming a prior Cauchy distribution with scale r = 0.707), which indicates moderate evidence in favour of the null hypothesis best explaining our data. Similarly, there was no significant difference in our planned contrast between the pleasant rating of Robot slow (M = .71, SD = .20) vs. Human slow touch (*M* = .75, *SD* = .18; t (42) = −.83, *p* = .416). We then conducted Bayesian analysis, testing the null hypothesis against the alternative hypothesis that robot touch may lead to less pleasant feelings than human touch. We obtained a result of BF_10_ = 0.475 (error%∼0.024, assuming a prior Cauchy distribution with scale r = 0.707), which suggests the evidence is inconclusive.

### 5.2 Relationship between STQ, pleasantness and activation

We anticipated a lower differentiation between CT-optimal and non-CT-optimal touch in people with generally more negative attitudes to social touch, irrespective of stimulation method. However, the results from the Social Touch Questionnaire were insignificant. Across stimulation type, the more the participant’s preference for social touch, the more they can discriminate between the pleasantness of slow and fast touch (r = −.416, *p* = .054), and the activation between fast and slow touch (r = −.453, **p = .034)**. Thus we did not find the total STQ score to be a significant predictor of the pleasantness ratings or of the activation ratings.

### 5.3 Changes in EEG band power due to different stimuli

Descriptive results are summarised in Table 1, including the average band power of five frequency bands (delta, theta, alpha, beta, and gamma) in all the brain regions (prefrontal, frontal, central, parietal, temporal, and occipital) during resting state and six types of tactile stimulus (fast and slow touch by brush, human and robot).

**TABLE 1:** Mean of band power in five different frequency bands in different brain regions for all types of stimuli.

		Pre-frontal	Frontal	Central	Parietal	Temporal	Occipital
Delta	Resting	156.04 (5.84)	153.12 (9.20)	152.47 (12.03)	155.18 (8.03)	159.56 (12.70)	151.72 (9.36)
	Human slow	156.52 (4.55)	153.54 (11.45)	153.82 (11.97)	164.31 (7.57)	163.49 (14.84)	159.38 (5.48)
	Brush slow	160.16 (4.62)	161.52 (13.33)	160.50 (13.86)	174.30 (8.65)	175.31 (17.08)	175.36 (7.47)
	S-CAT slow	155.84 (4.42)	154.59 (11.57)	152.11 (10.67)	164.94 (6.14)	163.87 (13.09)	160.81 (6.94)
	Human fast	158.81 (4.42)	157.87 (10.69)	155.66 (12.60)	165.63 (7.71)	167.03 (14.25)	165.37 (6.54)
	Brush fast	155.82 (4.76)	159.64 (14.19)	157.52 (15.15)	176.07 (9.07)	173.94 (20.20)	171.08 (7.65)
	S-CAT fast	156.07 (4.92)	153.41 (9.75)	153.69 (10.56)	161.40 (6.47)	162.33 (12.10)	159.46 (6.08)
Theta	Resting	98.69 (6.44)	100.35 (8.40)	97.40 (12.19)	104.35 (10.51)	105.26 (14.03)	101.21 (10.30)
	Human slow	98.50 (4.18)	101.46 (9.13)	98.45 (11.60)	112.29 (10.16)	109.02 (15.07)	108.25 (6.98)
	Brush slow	101.22 (3.94)	107.91 (12.73)	103.79 (15.07)	122.09 (10.09)	122.12 (18.27)	120.54 (10.37)
	S-CAT slow	98.29 (4.42)	102.02 (10.12)	96.78 (11.91)	113.21 (8.62)	111.84 (12.62)	108.59 (7.66)
	Human fast	100.45 (4.26)	104.53 (10.59)	100.20 (12.69)	113.51 (9.75)	113.63 (14.55)	113.83 (8.31)
	Brush fast	98.74 (4.71)	108.02 (15.64)	104.49 (17.37)	126.02 (11.06)	122.50 (22.05)	120.71 (10.78)
	S-CAT fast	99.31 (4.17)	100.90 (7.97)	97.50 (11.34)	109.19 (8.83)	107.40 (11.88)	104.78 (7.62)
Alpha	Resting	98.42 (6.29)	100.76 (8.22)	96.71 (10.17)	104.50 (9.65)	104.55 (13.19)	101.48 (10.03)
	Human slow	97.47 (3.62)	100.82 (7.92)	96.67 (9.57)	110.64 (8.90)	106.82 (12.67)	108.09 (6.25)
	Brush slow	99.55 (3.59)	106.11 (11.10)	101.35 (12.68)	118.68 (8.73)	118.36 (16.15)	116.06 (10.17)
	S-CAT slow	96.66 (4.44)	100.79 (9.03)	94.70 (9.68)	111.02 (8.31)	109.57 (11.01)	107.45 (7.36)
	Human fast	97.77 (3.97)	102.14 (9.54)	97.08 (10.18)	109.97 (8.26)	109.69 (11.95)	111.08 (7.37)
	Brush fast	93.96 (4.23)	102.64 (13.72)	98.45 (14.92)	119.21 (8.88)	116.05 (18.88)	113.02 (9.96)
	S-CAT fast	97.73 (4.44)	100.16 (9.03)	95.72 (9.68)	108.07 (8.31)	105.39 (11.01)	102.95 (7.36)
Beta	Resting	89.40 (6.14)	93.95 (9.72)	90.07 (11.45)	99.65 (11.27)	99.64 (15.89)	99.45 (10.62)
	Human slow	88.33 (2.84)	92.76 (8.45)	89.49 (9.93)	103.96 (9.74)	100.75 (14.16)	105.10 (6.19)
	Brush slow	88.91 (2.84)	96.61 (11.62)	93.00 (13.28)	111.18 (9.09)	110.84 (17.55)	110.19 (10.42)
	S-CAT slow	86.55 (3.60)	92.63 (9.62)	87.53 (10.73)	104.60 (8.39)	103.98 (12.35)	103.79 (6.72)
	Human fast	87.65 (3.50)	92.87 (10.13)	88.78 (10.95)	102.97 (8.79)	103.34 (13.13)	106.91 (7.11)
	Brush fast	83.52 (3.57)	93.00 (13.87)	89.25 (14.62)	109.70 (8.79)	107.27 (19.38)	104.67 (9.91)
	S-CAT fast	88.31 (3.59)	92.25 (7.67)	88.52 (9.90)	102.28 (8.42)	99.72 (11.28)	99.34 (6.26)
Gamma	Resting	88.30 (6.73)	95.37 (12.49)	89.87 (13.68)	103.18 (13.38)	102.19 (19.28)	106.38 (11.34)
	Human slow	88.08 (2.69)	93.20 (10.58)	88.60 (10.63)	105.76 (10.27)	103.32 (16.55)	111.45 (6.65)
	Brush slow	85.51 (2.19)	94.32 (12.56)	90.09 (14.53)	111.04 (10.13)	109.65 (19.00)	113.41 (9.93)
	S-CAT slow	83.64 (3.02)	92.28 (11.07)	85.99 (12.57)	106.83 (9.42)	106.03 (14.83)	110.35 (7.04)
	Human fast	86.69 (3.46)	92.36 (11.78)	87.43 (12.37)	106.05 (9.58)	105.84 (15.74)	113.55 (7.57)
	Brush fast	79.53 (3.30)	89.23 (12.56)	84.51 (14.53)	106.04 (10.08)	103.44 (19.98)	104.27 (10.04)
	S-CAT fast	85.45 (3.04)	91.09 (9.20)	86.34 (10.73)	104.15 (8.87)	101.98 (14.10)	105.31 (7.04)

A previous study (von Mohr et al., 2018) suggested an attenuation in the theta band power specific to the CT-optimal affective touch compared with the non-CT-optimal non-affective touch and resting state, particularly in the parietal and temporal regions. Here, a similar pattern was also observed in the beta band in the parietal region. Hence, in this study we compare the effect of three different CT and non-CT touch stimuli on the theta and beta band power in the parietal and temporal regions.

First, we compared the band power of all stimuli with the resting state in the theta and beta frequency bands in the temporal and parietal regions independently, using separate Friedman tests, as shown in Table 2, first row. We obtained significant differences in theta band power in the temporal (*p =* 0.001, χ2 = 23.26) and parietal (*p =* 0.007, χ2 = 17.88) regions and beta band power in the temporal region (*p =* 0.024, χ2 = 14.61) with different touch stimuli.

**TABLE 2:** Statistical results from analysing EEG data.

	Statistical test	Theta -temporal	Theta -parietal	Beta -temporal	Beta -parietal
1	Friedman’s test Rest-BF-BS-RF-RS-HF-HS	*p* = **0.001** *χ* ^2^ (6 df) = 23.26	*p* = **0.007** *χ* ^2^ (6 df) = 17.88	*p* = **0.024** *χ* ^2^ (6 df) = 14.61	*p* = 0.092 *χ* ^2^ (6 df) = 10.87
	Significant pairs from pairwise comparisons (*p* adjusted with Bonferroni correction)	Rest-BF *p* = **0.005**	Rest - BF *p* = **0.047**	—	—
2	Wilcoxon Signed Ranks Test to compare affective (slow) vs. non-affective (fast) touch				
	HS-HF	*p* = 0.091 *Z* = −1.689	*p* = 0.373 *Z* = −0.889	*p* = 0.286 *Z* = −1.067	*p* = 0.790 *Z* = −0.267
	BS-BF	*p* = 0.062 *Z* = −1.867	*p* = 0.075 *Z* = −1.778	*p* = 0.110 *Z* = −1.6	*p* = 0.328 *Z* = −0.978
	RS-RF	*p* = 0.155 *Z* = −1.423	*p* = 0.328 *Z* = −0.978	*p* = 0.213 *Z* = −1.245	*p* = 0.328 *Z* = −0.978
3	Friedman’s test to compare different stimuli for affective (slow) touch HS-BS-RS	*p* = 0.78 *χ* ^2^ (6 df) = 5.091	*p* = 0.234 *χ* ^2^ (6 df) = 2.9	*p* = 0.078 *χ* ^2^ (6 df) = 5.091	*p* = 0.086 *χ* ^2^ (6 df) = 4.909

*Values in bold are statistically significant.

HF, human hand fast speed; HS, human hand slow speed; BF, brush fast speed; BS, brush slow speed; RF, robot fast speed; RS, robot slow speed.

To investigate further, upon pairwise comparisons with Bonferroni correction, we observed that the band power of non-CT-optimal fast brush touch was significantly different from the resting state in theta in both the temporal (*p* = 0.005) and parietal (*p* = 0.047) regions. No other (affective and non-affective) touch stimuli showed a significant difference from the resting state.

Secondly, to assess differences in CT-optimal affective touch and non-CT-optimal non-affective touch for each stimulus, we compared band powers in pairwise human slow and human fast touch, brush slow and brush fast touch, and robot slow and robot fast touch conditions independently using Wilcoxon Signed Ranks Test for theta and beta frequencies in the temporal and parietal regions, as shown in Table 2, second row. We observed no statistical difference between CT-optimal affective and non-CT-optimal non-affective touch in EEG band powers, unlike the previous study.

Finally, to assess whether there are any differences in the band powers of different touch stimuli, we compared CT-optimal affective human, brush, and robot touch using a separate Friedman test for each theta and beta bands in the temporal and parietal region, as shown in Table 2, third row. We observed no statistically significant differences between affective touch from three types of stimuli, which shows promise for soft robotics as a mode of providing affective touch.

To summarise, in contrast to our hypothesis, we did not observe a decrease in theta and beta band powers between CT-optimal touch and resting state, and CT-optimal affective touch and non-CT-optimal touch, although as expected, there were no significant differences in the EEG band power of different types of stimulation (human, robot, brush).

### 5.4 Results from qualitative comments

The initial themes from the qualitative analysis included those that are common across all six experimental conditions, such as “accustomisation”, “associating physical attributes to characteristics of the stimulation” and describing the characteristics of the stimulations with “metaphor”; themes that are common across all three types of touch stimulators such as “slow touches felt more positive and fast touches more activating”; and themes that unique to one type of stimulator, such as that “ticklish” is associated with stimulation from brush, and “novelty” is associated with stimulation from the S-CAT device. We report the final themes developed below.

#### 5.4.1 Slow touches felt more positive and fast touches more activating

In general, participants reported that slow touch feels more comfortable or pleasant while fast touch feels more stimulating and less relaxing, irrespective of stimulation type (human hand, brush, or robot). For example, slow brush touch is commented as “quite pleasant and soft. Slowness is very relaxing, sleepy, calm. It is less arousing”, while fast brush touch is considered “less relaxing and more energising … less pleasurable”. Slow S-CAT touch is commented as “really relaxing”, “very soothing”, and “feel comfortable and safe”, while fast S-CAT touch is considered more “neutral”, “impartial”, and “alert (ing)”.

There were no major adverse emotions associated with slow robotic touch; instead, positive affective words dominated the qualitative comments. All three types of stimulations received overwhelmingly positive comments: there are 12 references for positive and pleasant comments compared with only 1 -2 references for negative comments for each stimulation type. Words such as “calming”, “soothing”, “relaxing”, and “pleasant” were used to describe all three stimulation types. On top of this, some participants assigned caring qualities to these slow touches. One participant referred to slow brush touch as “sensitive, careful”, one participant referred to slow human touch as “caring”, while three participants described slow S-CAT touch as “safe” and “reassuring”.

#### 5.4.2 Different characteristics reflected through metaphors regarding different tactile stimulations

There are differences in the attributes assigned by participants towards the three different types of stimulations. This can be observed through the metaphors used by the participants. Metaphors can provide insights into lived experiences of others, especially in the experience of touch, which can be difficult to express in words (Kelly et al., 2018). While participants associate human touch mostly with human touch, they assign various metaphors for the S-CAT haptic system and brush. For the S-CAT system, although novelty was expressed by some participants saying that they “can't associate with anything familiar”, more participants described it as “massage”, “blood pressure machine”, “human like”, “cuddle”, “water”, “wave” and “external creature”. For brush, participants described it as pets and animals, “feather”, “eyelash brushing”, and “fur”.

There is a distinctive difference in the metaphors given by the participants for slow and fast touch. For example, for human touch, slow touch is referred to as “ASMR”, “human hand”, “cat” and “velvet”, while fast touch is referred to as “a plant called ‘rabbit ear’“, “being painted”. For the S-CAT system, slow touch is referred to as “cuddling”, “massage”, “wave”, and “feel like human”, while fast touch is referred to as “heartbeat”, “impulse”, “electric shock”, “alarm clock”. For brush, slow touch is referred to as “my dog … so soft makes me sleepy and cuddly”, “my cat nestling next to me … showing me affection”, while fast touch is referred to as “playful cat”, “a naughty cat … wanting you to play with him or her, but you ignore him or her”, or “spider”. Slow brush touch was sometimes commented as ticklish, with the brushing direction from hand to elbow observed as more activating or even unpleasant, as it brushed against the direction of the hair growth. Some participants thus expressed their preference toward robot touch over brush touch as the touch from the haptic system does not irritate the skin hair.

#### 5.4.3 Design variables contributing to characteristics of tactile stimulation

Interestingly, some participants intuitively associate the felt quality of the stimulation with the properties of the three stimulation types. Repetition and familiarity were seen as contributing to the sense of comfort: “(slow S-CAT touch) feels really relaxing … maybe it is because of the repetitive movement”, “(the sense of) care comes from the repetition” (regarding slow S-CAT touch). On the other hand, repetition can also be seen as mechanical, while variation in stimulation pattern felt more realistic: “The repetition makes me associate with machine, not with a person. It felt less living” (regarding fast brush touch). “(S) eems less human, as it was always the same. Human hand would have had more variety so that feels better and less mechanical” (regarding slow brush touch). “(S) ome variation in the intensity made it felt organic” (regarding slow S-CAT touch). “(T) he feeling of variability made it not like a machine but human touch” (regarding slow S-CAT touch). Temperature is commented as an important feature contributing to positive affect. “The warm temperature makes you feel comfortable and safe” (on slow S-CAT touch). “It felt caring and trustful because of the temperature, the right pressure and it is skin-like” (on slow human touch). Moisture level is also commented as a factor impacting the felt affective quality: “(because the) hand is dry. If the hand is sweaty, it would be really different (felt negative)”. The level of applied normal force can also shape the characteristic of a touch: “a bit more pressure (would lead) to the masculine level.” (slow S-CAT touch) – the participant indicated that if the applied force increased, the touch would have felt more masculine. Several participants mentioned that the sound of human touch (the friction of skin) also contributes to the comforting feeling.

#### 5.4.4 Perceived continuity and accustoming

All three types of stimulation received comments on the experience of accustoming - the intensity of sensation was found to wear out over time. For slow S-CAT touch, some participants commented on the novelty of the sensation at the beginning. However, as the stimulation was repeated during the session, they commented that the familiarity increased, and the sensation could be perceived as more pleasant. Regarding the perceived continuity, only 9 out of 21 participants gave a rating on the perceived continuity of the stroking, with a mean score of 6.18 (SD1.25) out of 10, others preferred to give qualitative comments. Some participants referred to this touch as “stroking” while some others expressed that the perceived continuity could increase as the experience continues: “I felt the progression (the progression of the progressing cell pressuring onto the skin) at the start, when it goes on it (it reduced). At the last one, I felt smooth and don't feel the break anymore.”

#### 5.4.5 Robot touch can be preferable over human touch

Distinctively, when analysing comments towards all touch stimulations from a single participant, for the two participants who have an aversion towards being touched by another human (with high Social Touch Questionnaire Score of 47 & 49), acceptance was expressed towards touch from the S-CAT device. One participant expressed that slow touch from the human hand was “awkward” and felt “annoyed to be touched” while fast touch from the human hand felt “tickling like the skin is worn out, bleached and sting”; slow touch from the brush felt “uncomfortable, sting and hurt a bit”; she “prefer(s) robot/machine” and found fast touch from the S-CAT device “a bit calm”. The other participant who claimed, “not liking being touched by another human”, wanted to “resist” slow touch from the human hand, and found fast touch from human hand made her “nervous”. She found fast touch from brush “ichy” but found slow touch from the S-CAT device “comfortable, more acceptable”.

## 6 Discussion

### 6.1 Synthesis of results: relationship between STQ, pleasantness and activation

Both subjective ratings and qualitative comments revealed that, in line with our hypothesis, CT-optimal touch was perceived as more pleasant on average, while fast, non-CT-optimal touch was perceived as more stimulating on average, both irrespective of stimulation type (human hand, brush, or S-CAT system). There was no main effect of stimulation mode, nor the interaction between stimulation mode and velocity, in either measure. There was no significant difference between the pleasant ratings of slow touch delivered by the S-CAT system and brush, nor between the S-CAT system and the human hand. Bayesian analysis showed inconclusive results comparing slow touch delivered by the S-CAT system and human touch, however, it suggested moderate evidence supporting that there is no difference in pleasantness rating between slow touch delivered by the S-CAT system and brush. Qualitative comments suggested a similar level of positivity toward slow touch from the robot, brush, and human hand. In the EEG responses, there were no significant differences in the EEG band power of different types of stimulation, suggesting that slow robot touch, as delivered by our wearable device, can produce comparable pleasantness to that of slow brush and slow human touch, although these EEG effects should be treated with caution as we were not able to confirm previous findings of velocity modulation even within modalities. Indeed, contrary to part of our hypothesis, we did not observe a decrease in theta and beta band powers between CT-optimal affective touch and resting state, and CT-optimal affective touch and non-affective, non-CT-optimal touch. This could be due to our small sample size and limited number of EEG trials sample, or it may relate to the fact that CT-optimal touch may not be associated with a clear pattern of EEG effects as suggested by the conflicting results of previous studies (see 4.1). Nevertheless, the confirmation regarding the subjective effects of slow (vs. fast) robotic touch is an encouragement for future work to improve the S-CAT device, while its neurophysiological effects may be best captured in future studies by other functional neuroimaging techniques such as magnetic resonance imaging (The feature that the wearable elements of the S-CAT system is free from any metal or magnetic component makes it fit to be used in an fMRI equipment).

### 6.2 Insights in simulating human stroking touch and opening-up design space

Our adoption of the combination of parameters of stroking velocity (6 cm/s), applied normal force (0.48N) and temperature (slightly warmer than skin temperature) turned out to be effective in producing affectively positive touch stimulations. There was a consensus among participants’ responses that the slow stroking velocity felt more pleasant and relaxing than the fast velocity. Especially, the temperature is most frequently commented as contributing to the feeling of sensorial pleasantness (“feel comfortable”) but also leads to the affect of a social nature such as feeling “safe” and “reassuring”, which agrees with Nie et al. (2012) and Block and Kuchenbecker (2019) in which participants perceived “trust” when touched by a robotic agent that has warmth. From our participants’ qualitative comments, we have learned several insights on the design variables that contribute to higher fidelity of human affective touch. More variations in stimulation patterns and applied normal force levels contributes to the perception of being more human-like. The continuity of the stroke of the “rippling” mechanism of the S-CAT can be further improved. It is worth noting that moisture in the contacting surface negatively contributes to a positive affect. The sound, instead of noise, can have a positive value. Repetition and familiarity can contribute to the relaxing quality but can also lead to less human-like and more mechanical quality. Flow rate, that is, the amount of air going into each actuator cell within a given time, impacts the felt quality of the stroking. This was found during the iterative development of the S-CAT system. For the same sized actuator cell, a bigger flow rate results in faster speed in reaching the maximum force onto the skin, which reflects a more forceful quality of stroking, while a smaller flow rate results in slower speed in reaching the maximum force onto the skin, which reflects a gentler quality of stroking. A faster speed of reaching the maximum force of each actuator cell also results in a less continuous feeling of the stroking. In a recent review, Raisamo et al. (2022) pointed out that exploiting the opportunity of haptic illusions offers a great opportunity to make haptic communication more expressive in supporting social and affective communication, however currently there is a lack of studies exploring the affective aspects of illusions. Existing work explored such potential in voice coil actuators (Culbertson et al., 2018; Nunez et al., 2020) and Shape Memory Alloy actuators (Muthukumarana et al., 2020). Our study responds to this challenge and adds to this body of knowledge by evidencing the affective quality of a pneumatic actuator with a closer distance of adjacent indentation force than previous work. The qualitative comments from participants point to that the perceived sense of continuity could increase over time as the experience continues, a phenomenon that wasn’t observed in above-mentioned studies. Future work on pneumatic devices exploiting tactile illusion for stroking affective touch similar to the S-CAT device can include testing different combinations of duration, inflation and deflation speed, time of delay, and their relation to the perceived continuity. Although the S-CAT received a high level of rating on pleasantness comparable to that elicited by an established CT-optimal touch stimulator (soft brush), further study is needed to evaluate if the approach of the haptic illusion of stroking does indeed stimulate CT-afferents.

### 6.3 Design space and the ethics of mimicking human touch

The qualitative comments gave interesting information to inspire design opportunities for touch-based robotic entities to participate in therapeutic and care applications. Slow human touch was perceived with the most consistency to be pleasant, comfortable, and relaxing, although two participants reported an aversion to human touch, and one participant attributed such touch aversion to the experience of social touch with a negative affective valence in her childhood. This aversion may be linked to findings from studies on social anxiety contributing to aversion toward inter-personal affective and supportive social touch (Kashdan, Doorley, Stiksma and Hertenstein, 2017; Krahé et al., 2018; Lapp and Croy, 2020; Wilhelm et al., 2001). Interestingly, two participants who considered human touch to be psychologically uncomfortable, found both slow and fast robot touch more acceptable than human and brush touch. Wilhelm et al. (2001) also pointed out that although people with high social anxiety can have very different acceptance of human touch than those without, they share a similarity in their acceptance of non-human touch that does not link with a social intention as in inter-personal touch. This raises an interesting tension, as while haptic design research explores means to achieve better social quality in touch initiated from technology, this feedback seems to suggest that a lack of social quality in affective touch from technologies can be desirable in such a context. This on one hand illuminates a design space in which for individuals who are prevented from benefiting from social and affective touch due to touch aversion, an affective touch performed by a wearable S-CAT device can be a helpful alternative, and on the other hand encourages further research on the desirable balances of social qualities in affective haptics suitable for different contexts. In addition, future studies could explore whether in certain situations, such as clinical settings, where there are subtle boundaries between professional touch, comforting touch, and inappropriate touch (Bruhn, 1978), an S-CAT soft robotic device to comfort patients in distress may be helpful to reduce the complexity of social connotations. The concept of an S-CAT device can also enable remote affective touch on occasions when these touches are inaccessible, for example in intensive care, in telecare, in treatment that requires patients to be in isolation such as in radiotherapy (Goldsworthy et al., 2020), or during quarantine amid a pandemic such as COVID-19.

However, speculating on such design space could not be done without care in considering ethical implications. Research in this domain has particularly advocated for due attention given to ethical considerations when introducing robotics that interact through tactile modality. It has been pointed out that humans and mammals can develop affective attachments to objects, our tools, and robots (Scheutz, 2011), and that compared with hard-bodied robotic agents, soft-bodied robots have a stronger conduit for bonding (Arnold and Scheutz, 2017). In our study, some participants indeed associated the S-CAT touch with human touch, and even endowed it with a caring quality, which indicates a relational quality. A machine touch that mimics human touch can invite affective bonding, either intended or unintended. While the above-speculated design opportunities assume it beneficial for users having to cope with stressful scenarios alone to be assisted by robotics with affective touch capability, other dimensions of relational impact such as unintended bonding remain unknown and require investigation. Such investigation requires the direct involvement of users and taking individual and contextual elements into consideration (Jewitt et al., 2021). In addition, research on the appearance of robots has shown that robots that look almost like real humans evoke discomfort in humans, the so-called “uncanny valley” effect (Mori, 2012). Recent research indicates that this “uncanny valley” issue may also apply to robot touch (D’Alonzo et al., 2019). It is worth investigating whether it is true that the more a machine-delivered touch feels like a real human touch, the more positive affect it enables.

### 6.4 Limitation

The limitations of the study also include the fact that only two velocities were tested, and the effects of the experimenter’s gender were not examined, nor were the sexual orientation of our participants. The study further relied on subjective, rather than behavioural measures, and hence social desirability biases cannot be excluded. Finally, this was a lab-based study and thus robotic touch will eventually need to be evaluated in everyday life.

## 7 Conclusion

In this study, motivated by the promising affective qualities of haptic sensation from soft robotics, and the social-emotional benefit of CT-optimal affective touch, we developed S-CAT, a haptic rendering system to provide CT-optimal affective touch, with temperature and applied force informed by literature. We present the design and evaluation of the S-CAT system. We compared subjective ratings on pleasantness and intensity, neurophysiological responses, and qualitative comments about touch stimulation at CT-optimal and non-CT-optimal speeds, performed by an S-CAT device (robot touch), skin-to-skin touch (human touch) and a widely used affective touch stimulator (brush touch). The results suggested that the newly developed S-CAT device can deliver touch at different velocities, and slow velocities can lead to stronger feelings of tactile subjective pleasantness than fast velocities, as in skin-to-skin touch and brush stroking. The results also suggested that affective touch delivered by the S-CAT device provides comparable pleasantness to that elicited by brush stroking. The study detected no statistical differences in neurophysiological responses, but the combination of quantitative and qualitative behavioural results from our sample warrants further investigation into the potential benefits of the S-CAT device as a device for the exchange of remote, affective touch. We reflected on the insights gained through the study toward designing higher fidelity of human affective touch. We also reflected on the design space this study illuminates—the exploration of which requires careful ethical considerations. Future work could include the validation of the psychological benefit of touch stimulation from this system with a larger sample size, as well as investigating if similar psychological benefit can be achieved when applying stroking touch at varied speeds and applied force levels, and to other parts of the body.

## Data Availability

The original contributions presented in the study are included in the article/Supplementary Material, further inquiries can be directed to the corresponding author.

## Supplementary material

SUPPLEMENTARY DATA SHEET S1
Social touch questionnaire.
