# Supplementary material for: Simulating the psychological and neural effects of affective touch with soft robotics: an experimental study
Source: Front Robot AI. 2024 Nov 29;11:1419262. doi: 10.3389/frobt.2024.1419262 (PMC11638054; doi:10.3389/frobt.2024.1419262)
Supplement: Supplementary file 1 [file DataSheet1.pdf]

contact: yan.zheng@network.rca.ac.uk

Social Touch Questionnaire *

Participant No. __________

Date: __________________

		not at all 0	slightly 1	moderately 2	very 3	extremely 4
1	I generally like when people express their affection towards me in a physical way (R)					
2	I feel uncomfortable when someone I don't know very well hugs me					
3	I get nervous when an acquaintance keeps holding my hand after a handshake					
4	generally seek physical contact with others (R)					
5	I feel embarrassed if I have to touch someone in order to get their attention					
6	I consider myself to be a 'touchy-feely' person (R)					
7	It annoys me when someone touches me unexpectedly					
8	I'd feel uncomfortable if a professor touched me on the shoulder in public					
9	I'd be happy to give a neck/shoulder massage to a friend if they are feeling stressed (R)					
10	I feel uncomfortable if I make physical contact with a stranger on the bus or subway					
11	I like being caressed in intimate situations (R)					
12	As a child, I was often cuddled by family members (e.g. parents, siblings) (R)					
13	I would rather avoid shaking hands with strangers					
14	I greet my close friends with a kiss, cheek-to-cheek (R)					
15	I feel comfortable touching people I do not know very well (R)					
16	I feel disgusted when I see public displays of intimate affection					
17	It would make me feel anxious if someone I had just met touched me on the wrist					
18	If I had the means, I would get weekly professional massages (R)					
19	I hate being tickled					
20	I like petting animals					

*Wilhelm, F. H. et al. (2001) Social anxiety and response to touch: incongruence between self-evaluative and physiological reactions. *Biological Psychology*. [Online] 58 (3), 181–202.

*Wilhelm, F. H. et al. (2001) Social anxiety and response to touch: incongruence between self-evaluative and physiological reactions. *Biological Psychology*. [Online] 58 (3), 181–202.
